# The diagnostic performance of dual-layer spectral detector CT for distinguishing breast cancer biomarker expression and molecular subtypes

**DOI:** 10.1038/s41598-024-51285-3

**Published:** 2024-01-17

**Authors:** Lanjing Chen, Zhengyuan Xiao, Jianmei Fu, Jingrong Huang, Yongshu Lan

**Affiliations:** 1 Department of Radiology, The Affiliated Hospital, Southwest Medical University, Luzhou, China; 2 Southwest Medical University, Luzhou, China

**Keywords:** Breast cancer, Outcomes research

## Abstract

To evaluate the diagnostic performance of dual-layer spectral detector CT for differentiation of breast cancer molecular subtypes. This study was done in a retrospective approach including 104 female patients histopathologically proven to have breast cancer. These patients underwent chest arterial and venous phase dual-layer SDCT. CT values, iodine concentrations (IC)s, and Z-effective (Zeff) values of the lesions and arteries in the same layer were determined for both arterial and venous phases. Parameter values were normalized, and slopes of the spectral curves (λHu) were calculated. Breast cancer biomarkers were also analyzed. Afterward, correlations between the obtained parameters and biomarkers were analyzed. Eventually, the diagnostic performance was assessed using ROC curves. ER or PR-negative patients generally showed significantly higher mean iodine concentrations, CT, and Z-effective values. HER2-positive patients showed significantly higher CT_VE_, Zeff_VE_, N-Zeff_VE_, IC_ART_, IC_VE_, NIC_ART_, NIC_VE_, and λ_VE_. Only IC_VE_ and Zeff_VE_ differed significantly between Ki67-positive and negative patients. All parameters showed significant diagnostic value for subtypes except N-Zeff_ART._ Luminal and non-luminal types differed significantly and ROC curves indicated that multi-factors had the best diagnostic efficacy. The dual-layer SDCT distinguishes breast cancer biomarker expression and molecular subtypes. Thus, it can be used for preoperative assessment of breast cancer.

## Introduction

Breast cancer is a common malignancy and since 2020 is responsible for the majority of female cancer-associated deaths^1,2^. Both the incidence and mortality of the disease are increasing, with increased prevalence in younger patients^3^. Although the pathogenesis is not fully understood, risk factors include genetics, hormone therapy, pregnancy-associated factors, and poor lifestyle, among others^4,5^.

The eighth edition of AJCC Cancer Staging emphasized the value of immunohistochemical markers for the diagnosis of breast cancer. For example, high expression of ER (estrogen receptor) and PR (progesterone receptor) suggest that the patient will respond to endocrine therapy, while HER2 (human epidermal growth factor receptor-2) positivity is associated with unfavorable prognosis. Breast cancer is further classified according to biomarker expression into Luminal A, Luminal B, HER2-overexpression, and triple-negative subtypes^6^. These subtypes vary in aggressiveness and efficacy of treatment^7^.

Immunohistochemistry is the gold standard for the clinical diagnosis of breast cancer molecular subtypes. However, this is an invasive examination and may lead to complications. In addition, the biopsy tissue used for immunoassays may not fully reflect the overall tumor type due to tumor heterogeneity^8^. The use of imaging examinations overcomes the heterogeneity of tumor tissues in both time and space and has been found to have sensitivity and specificity for breast cancer diagnosis. Dual-layer SDCT is the latest embodiment of energy spectrum technology that reduces the radiation dose to the patient and provides high and low energy datasets that are fully registered in space and time, thus improving temporal resolution and reducing noise in the spectral images^9^. It has been found^10,11^ that spectral CT has specific diagnostic value for evaluating the histological type, grade, and staging of breast tumors. To the best of our knowledge, studies on molecular subtypes of breast cancer are limited. Therefore, we intended to explore the diagnostic performance of dual-layer SDCT for distinguishing breast cancer molecular subtypes without increasing the economic burden of patients.

## Materials and methods

### Patients

The clinical and imaging data of patients with breast cancer who underwent dual-layer SDCT scans at the Affiliated Hospital of Southwest Medical University, China, between January and July 2021 were retrospectively analyzed. The inclusion criteria comprised histopathological diagnosis of breast cancer, no prior treatment in the breast, single breast lesion per breast and satisfactory image quality for the concerned study, the maximum diameter larger than 10 mm. Alternatively, the exclusion criteria were lack of histopathological diagnosis of breast cancer, missing patients’ clinical or imaging data, multiple lesions in a single breast, prior breast treatment and poor image quality. The included patients gave written informed consent to participate in the study. The study received ethical approval from the Ethics Committee of the Affiliated Hospital of Southwest Medical University (KY2022160), and strictly followed the guidelines of the Declaration of Helsinki.

### Scanning technique

All patients underwent dual-phase enhanced scans of the chest using the dual-layer SDCT with the following scan parameters: 120 kVp; automatic mAs technology; rotation time, 0.4; pitch, 1.375; collimation 64 × 0.625 mm; slice thickness, 1 mm; increment, 1 mm. The midsagittal plane of the body perpendicular to the examination table and coinciding with the midline of the long axis of the examination table, and scanning range from supraclavicular to subdiaphragmatic. A total of 65–90 ml (1.2 ml/kg) contrast agent (Ioversol 320) was administered through the antecubital vein at a rate of 3 ml/s. This was followed by 20 ml of saline injected at the same rate, and 40 ml of saline were injected after administration of the contrast agent. The enhanced scanning adopted the threshold automatic triggering technology, using bolus tracking in the aortic arch with the threshold set at 200 Hounsfield units (HU). Arterial phase scanning began on reaching 200 HU, and the venous-phase images were obtained after a 30-s delay after the arterial phase scan.

### Postprocessing and image analysis

**Figure 1:**
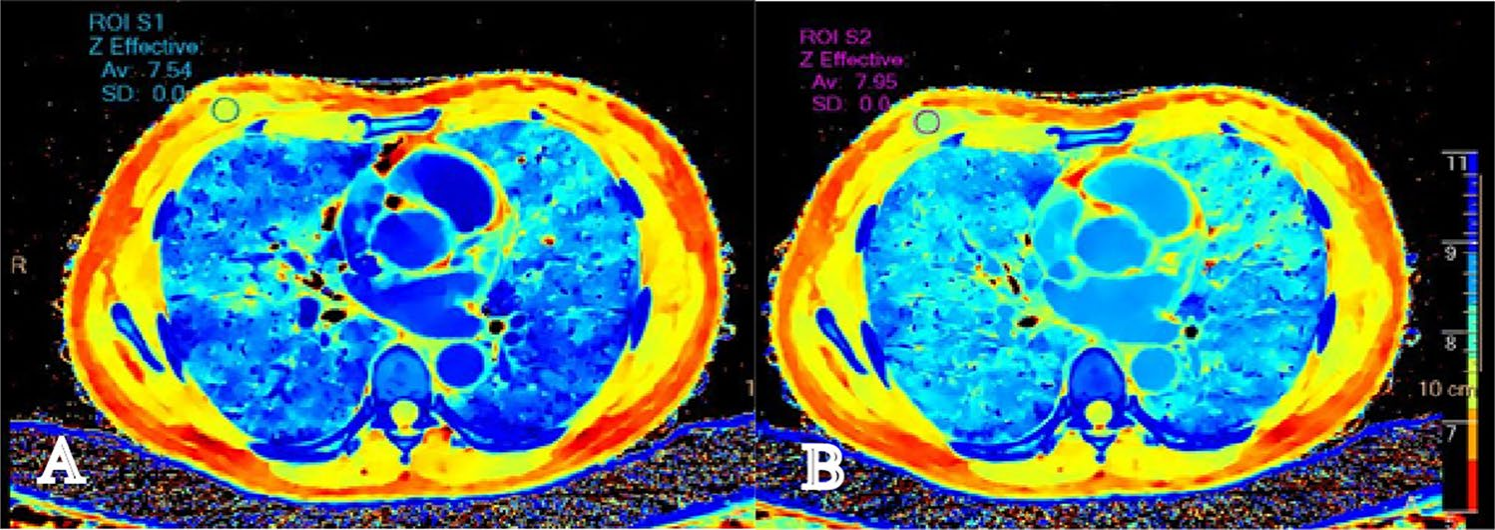
Z-effective. The average Z-effective values in the arterial phase (**A**) and venous phase (**B**) is 7.54, 7.95 respectively, and the lesion and surrounding glandular tissue can be clearly distinguished in venous phase images.

**Figure 2:**
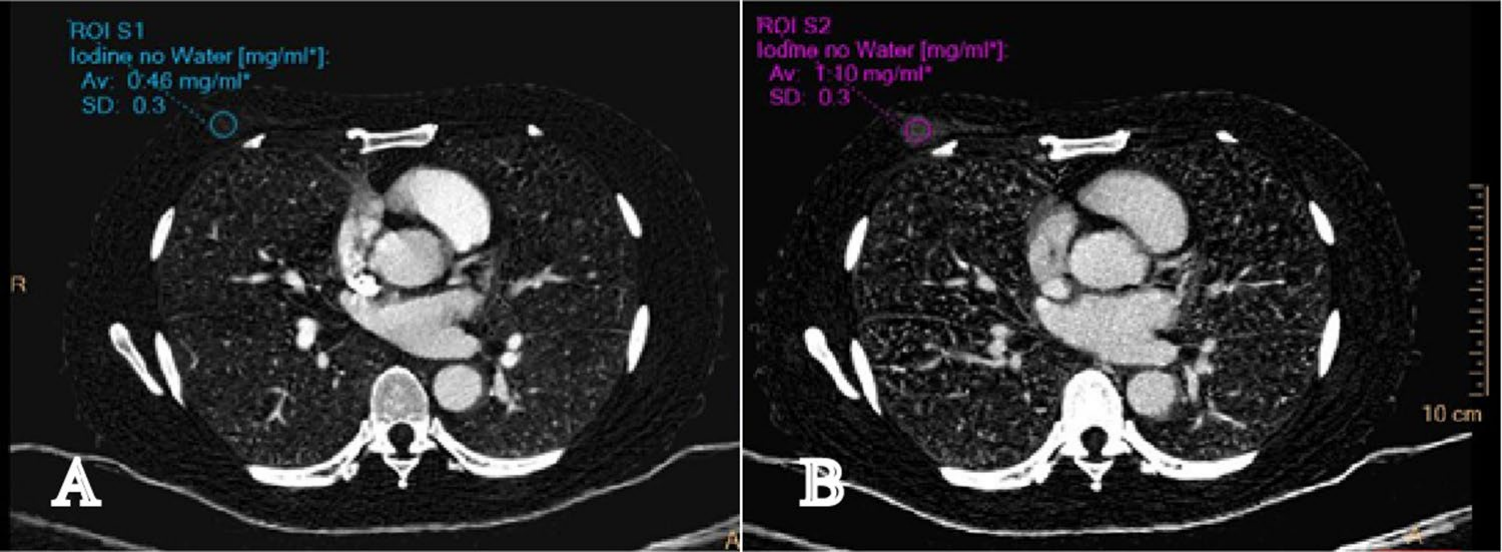
Iodine no water. The average iodine values in the arterial phase (**A**) and venous phase (**B**) is 0.46 mg/ml, 1.10 mg/ml, respectively.

**Figure 3:**
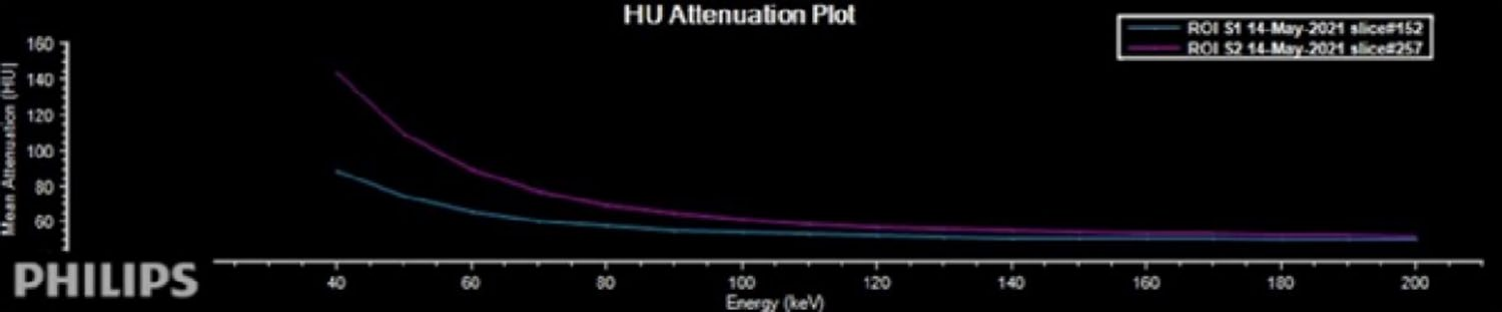
Spectral curve ROI-S1 and ROI-S2 are the spectral curves generated based on the ROI area of the lesions in the arterial and venous phases respectively. It can be seen from the figure that the curves gradually become flat after 100 keV.

Spectral based images (SBIs) can be automatically generated for every patient undergoing spectral CT scanning, without the need for parameter adjustments prior to the scan. Following the scan, a range of spectral images can be reconstructed and quantified using the Philips imaging workstation, with the entire process being accomplished within 3–5 min. All images were selected for reconstruction with conventional mixed energy images (CIs) and SBIs by Spectral Diagnostic Suite software (v 6.5.3) with both the reconstruction slice thickness and the increment being 1 mm. The Z-effective-maps (Fig. 1), iodine no water-maps (Fig. 2), and the virtual monoenergetic-images (VMI) at 40 and 80 keV were obtained from the arterial and venous phase images, respectively. The region of interest (ROI) was selected interactively by two radiologists each with over 4 years’ experience who were blinded to the patient’s clinical information and final diagnosis. The ROI was marked on the lesion area as well as in the aorta of the same layer on the venous phase images of CIs. The ROIs were then copied semi-automatically onto the arterial phase images of the CIs, the iodine density and Zeffective maps, and the VMI40 and the VMI80 to ensure consistency in the size, shape, and position between the different images. The ROIs were drawn as extensive as possible to include most of the enhanced part of the mass. Cystic, necrotic, and calcified regions in masses with mixed characteristics were carefully avoided. ROIs of 25–100 mm^2^ were placed at the level of the maximum diameter of the tumor. No tumors below 25 mm^2^ were observed in this study. The ROIs of the arteries were placed in the center of the lumen avoiding the calcified part of the blood vessel. The standardized value of each parameter was calculated using the formula: Normalized value = value of lesion/value of artery. The spectral curve of the ROI area of the lesion (Fig. 3) was generated by the software, and the slope value of the spectral curve was calculated according to the formula:

$$\lambda = ({\text{HU}}_{{80\;{\text{keV}}}} \, - {\text{ HU}}_{{40\;{\text{keV}}}} )/(80\;{\text{keV }} - \, 40\;{\text{keV}}).$$

### Histopathological analysis

The pathological results included both the histological type and immunohistochemical results. The immunohistochemical data included ER, PR, and HER2 positivity, as well as the cell proliferation index of Ki67. ER and PR expression rates of more than 1% were considered positive^12^. HER2-overexpression (grade 3+) or grade 2+ with a positive genetic test were classified as HER2-positive^3^. The Ki67 index was considered positive when the Ki67 expression rate was 20% and above^7^.

### Statistical analysis

All statistical analyses were conducted using SPSS 25.0 and MedCalc. The Kolmogorov–Smirnov test was used to measure the distribution type of quantitative data. If the data satisfied the normal distribution criteria, they were expressed as mean ± SD, while non-normally distributed data were expressed as median ± Interquartile range. Independent-samples *t*-tests and Mann–Whitney *U* tests were used to evaluate differences between different biomarkers expression groups. Kruskal–Wallis *H* test and ANOVA were performed to compare differences in spectral parameters between different molecular subtypes, and paired tests by using the LSD test. Thirty cases were randomly selected from the 104 patients for consistency analysis, and intra- and inter-observer agreement were evaluated by the intraclass correlation coefficient (ICC) and 95% confidence interval (95% CI). Good intra- and inter-observer agreements were observed and the reproducibility of the measurements was higher when the value of ICC > 0.75. Statistical significance was defined as *P* < 0.05.

## Results

### Consistency evaluation

Good intra-observer and inter-observer agreements were found and the measurement reproducibility was high (Table 1).

**Table 1:** Analysis of ICC value within and between groups of CT value of venous phase of lesions and artery.

Group	*P* value	ICC	95% CI
Intra-reader (lesions)	0.001	0.992	0.983–0.996
Inter-reader (lesions)	< 0.001	0.990	0.979–0.995
Intra-reader (vessels)	< 0.001	0.975	0.949–0.988
Inter-reader (vessels)	< 0.001	0.846	0.702–0.924

### Patient information

A total of 104 female (age range 31–86 years, mean age of 52.79 ± 9.27 years) with 105 lesions were included, including one case of a bilateral single mass, 92 were invasive carcinoma of unspecified type (87.6%), four were ductal carcinoma in situ (3.8%), eight were invasive carcinoma with ductal carcinoma in situ (7.6%), and one was mucinous carcinoma (1%). No tumors below 25 mm^2^ were observed in this study.

### Immunohistochemical markers

Except for NCT_ART_, NCT_VE_, and N-Zeff_ART_, the other spectral parameters differed significantly between ER- and PR-negative and positive patients (*P* < 0.05), with higher values in the ER- and PR-negative cases. The differences in these parameters (CT_VE_, Zeff_VE_, N-Zeff_VE_, IC_ART_, IC_VE_, NIC_ART_, NIC_VE_, and λ_VE_) between HER2 negative- and positive-patients were statistically significant, with higher values in the positive cases (*P* < 0.05). Only IC_VE_ and Zeff_VE_ showed significant differences between Ki67-negative and positive patients (*P* < 0.05), with higher values in positive patients (Table 2).

**Table 2:** Comparison results of spectral parameters with breast cancer prognostic biomarkers.

*N* = 105	CT_ART_	NCT_ART_	CT_VE_	NCT_VE_	Zeff_ART_	N-Zeff_ART_	Zeff_VE_
ER (*p*)	0.000^b^	0.061^b^	0.000^b^	0.002^b^	< 0.001^a^	0.169^b^	< 0.001^b^
P (*N* = 67)	50.20 ± 8.30	0.18 ± 0.06	72.10 ± 13.80	0.51 ± 0.09	7.52 ± 0.12	0.68 ± 0.05	7.88 ± 0.25
N (*N* = 38)	57.65 ± 12.20	0.19 ± 0.06	81.60 ± 15.95	0.57 ± 0.15	7.69 ± 0.19	0.69 ± 0.05	8.09 ± 0.26
PR (*p*)	0.007^b^	0.186^b^	0.023^b^	0.114^b^	< 0.001^b^	0.313^b^	< 0.001^b^
P (*N* = 76)	51.30 ± 9.33	0.18 ± 0.06	72.50 ± 15.77	0.52 ± 0.10	7.52 ± 0.17	0.68 ± 0.04	7.89 ± 0.24
N (*N* = 29)	57.70 ± 12.85	0.19 ± 0.07	78.70 ± 15.60	0.55 ± 0.16	7.64 ± 0.25	0.68 ± 0.05	8.10 ± 0.22
HER2 (*p*)	0.128^a^	0.119^b^	0.006^b^	0.135^b^	0.110^b^	0.459^b^	0.002^b^
P (*N* = 34)	55.84 ± 8.40	0.20 ± 0.06	78.70 ± 12.78	0.55 ± 0.13	7.59 ± 0.27	0.69 ± 0.05	8.05 ± 0.23
N (*N* = 71)	52.37 ± 9.46	0.18 ± 0.06	72.10 ± 16.20	0.53 ± 0.11	7.56 ± 0.18	0.68 ± 0.04	7.89 ± 0.22
Ki67 (*p*)	0.108^a^	0.55^b^	0.246^a^	0.458^a^	0.175^b^	0.675^b^	0.044^b^
P (*N* = 90)	54.09 ± 8.56	0.18 ± 0.05	74.30 ± 10.66	0.54 ± 0.09	7.56 ± 0.22	0.68 ± 0.04	7.97 ± 0.26
N (*N* = 15)	49.95 ± 12.31	0.18 ± 0.14	68.64 ± 17.67	0.51 ± 0.18	7.48 ± 0.22	0.68 ± 0.08	7.86 ± 0.36

*N* = 105	N-Zeff_VE_	IC_ART_	N-IC_ART_	IC_VE_	N-IC_VE_	λ_ART_	λ_VE_
ER (*p*)	< 0.001^b^	< 0.001^b^	< 0.001^b^	< 0.001^a^	< 0.001^a^	< 0.001^b^	< 0.001^b^
P (*N* = 67)	0.87 ± 0.03	0.44 ± 0.30	0.04 ± 0.03	1.03 ± 0.37	0.28 ± 0.10	0.70 ± 0.53	1.72 ± 0.58
N (*N* = 38)	0.89 ± 0.05	0.62 ± 0.52	0.07 ± 0.05	1.38 ± 0.33	0.37 ± 0.09	1.00 ± 0.69	2.21 ± 0.53
PR (*p*)	0.008^b^	0.002^b^	0.004^b^	0.004^a^	0.006^a^	0.003^b^	0.017^b^
P (*N* = 76)	0.87 ± 0.04	0.45 ± 0.31	0.04 ± 0.03	1.09 ± 0.39	0.29 ± 0.10	0.75 ± 0.54	1.81 ± 0.60
N (*N* = 29)	0.89 ± 0.05	0.60 ± 0.44	0.06 ± 0.05	1.33 ± 0.34	0.36 ± 0.10	0.99 ± 0.77	2.13 ± 0.57
HER2 (*p*)	0.005^b^	0.045^b^	0.028^b^	0.013^a^	0.014^a^	0.097^b^	0.033^b^
P (*N* = 34)	0.89 ± 0.04	0.54 ± 0.45	0.06 ± 0.05	1.29 ± 0.39	0.35 ± 0.10	0.93 ± 0.76	2.08 ± 0.65
N (*N* = 71)	0.87 ± 0.04	0.47 ± 0.33	0.04 ± 0.03	1.09 ± 0.38	0.29 ± 0.11	0.75 ± 0.55	1.81 ± 0.57
Ki67 (*p*)	0.103^b^	0.145^b^	0.241^b^	0.013^a^	0.118^a^	0.115^b^	0.152^b^
P (*N* = 90)	0.87 ± 0.03	0.50 ± 0.36	0.05 ± 0.04	1.19 ± 0.37	0.32 ± 0.10	0.85 ± 0.63	1.93 ± 0.60
N (*N* = 15)	0.86 ± 0.06	0.38 ± 0.41	0.03 ± 0.06	0.92 ± 0.45	0.25 ± 0.14	0.53 ± 0.62	1.69 ± 0.67

*P* positive, *N* negative, *N-CT* normalized CT values, *N-Zeff* normalized Z-effective, *N-IC* normalized iodine concentration. ^a^using *t-*test, ^b^using Mann–Whitney *U* test.

### Molecular subtypes

All spectral parameters showed higher levels of diagnostic efficacy for breast cancer molecular subtypes except N-Zeff_ART_ (Table 3). In the present study, spectral parameter values differed significantly between the Luminal and non-Luminal types (*P* < 0.05), particularly, between LA versus non-Luminal and Luminal versus triple-negative types. However, there were no significant differences between the Luminal A and Luminal B nor between the HER2-overexpression and triple-negative types (*P* > 0.05).

**Table 3:** Comparison results of spectral parameters with breast cancer molecular subtypes.

Type	LuminalA	LuminalB	N-HER2	TNBC	*P*
CT_ART_	45.37 ± 13.13	52.13 ± 7.27	59.57 ± 9.09	57.75 ± 10.91	0.001^a^
CT_VE_	60.59 ± 19.52	72.88 ± 10.62	79.62 ± 10.64	76.73 ± 10.80	0.004^a^
NCT_ART_	0.18 ± 0.16	0.18 ± 0.06	0.22 ± 0.08	0.19 ± 0.05	0.049^b^
NCT_VE_	0.42 ± 0.17	0.52 ± 0.09	0.62 ± 0.17	0.56 ± 0.15	0.008^b^
Zeff_ART_	7.39 ± 0.27	7.52 ± 0.16	7.70 ± 0.50	7.62 ± 0.24	0.001^b^
Zeff_VE_	7.67 ± 0.55	7.94 ± 0.22	8.13 ± 0.13	8.01 ± 0.23	0.001^b^
N-Zeff_ART_	0.68 ± 0.08	0.68 ± 0.04	0.71 ± 0.06	0.68 ± 0.04	0.257^b^
N-Zeff_VE_	0.83 ± 0.07	0.87 ± 0.04	0.90 ± 0.03	0.88 ± 0.04	0.001^b^
IC_ART_	0.34 ± 0.24	0.48 ± 0.22	0.73 ± 0.38	0.68 ± 0.32	0.001^a^
IC_VE_	0.70 ± 0.88	1.13 ± 0.38	1.46 ± 0.54	1.20 ± 0.51	0.002^b^
NIC_ART_	0.02 ± 0.07	0.04 ± 0.03	0.07 ± 0.08	0.07 ± 0.05	0.012^b^
NIC_VE_	0.17 ± 0.11	0.30 ± 0.10	0.38 ± 0.10	0.35 ± 0.10	0.001^b^
λ_ART_	0.52 ± 0.37	0.78 ± 0.37	1.16 ± 0.70	1.15 ± 0.53	0.008^b^
λ_VE_	1.41 ± 0.65	1.85 ± 0.59	2.10 ± 0.75	2.13 ± 0.44	0.030^a^

*ART* arterial phase, *VE* venous phase, *N-HER2* HER2-overexpression, *TNBC* triple-negative breast cancer, ^a^using ANOVA test, ^b^using Kruskal–Wallis *H* test.

### Diagnostic efficacy

**Figure 4:**
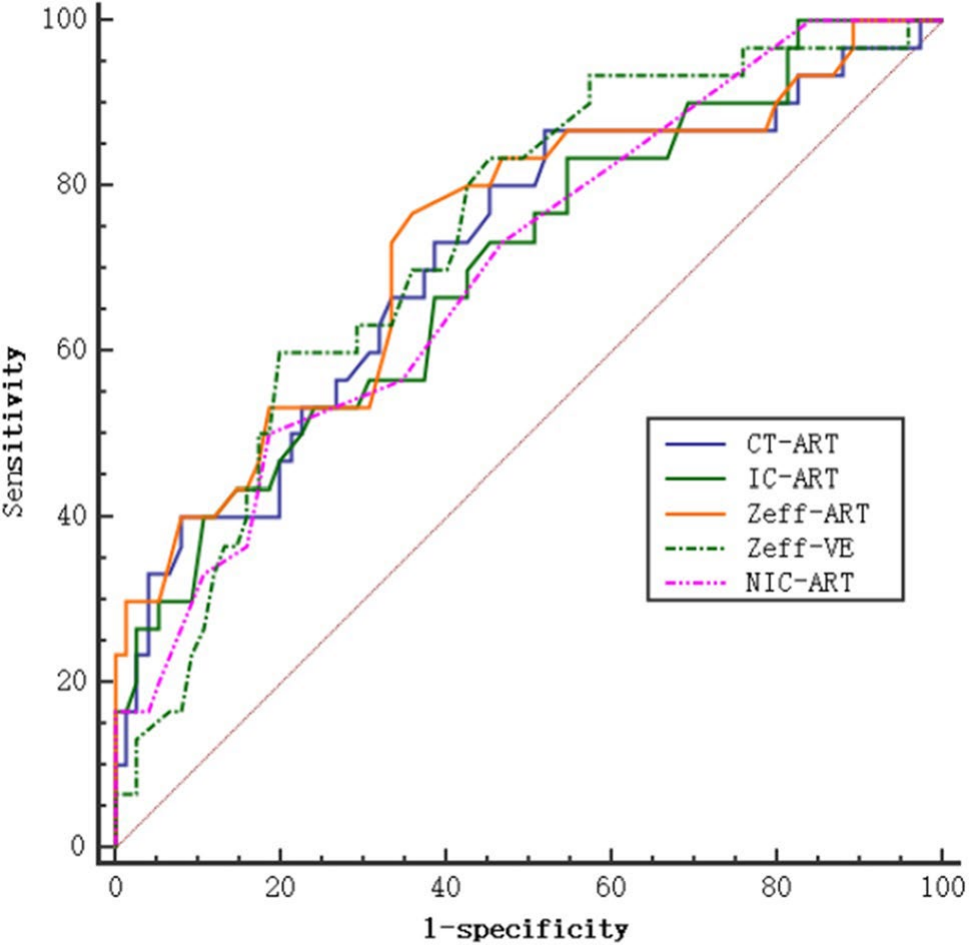
Comparison of area under the curve for diagnostic accuracy of spectrum parameters of lesions between Luminal type and non-Luminal type breast cancer. The diagnostic performance of Zeff_ART_, Zeff_VE_ was the best which the coordinate point was closest to the upper left.

**Figure 5:**
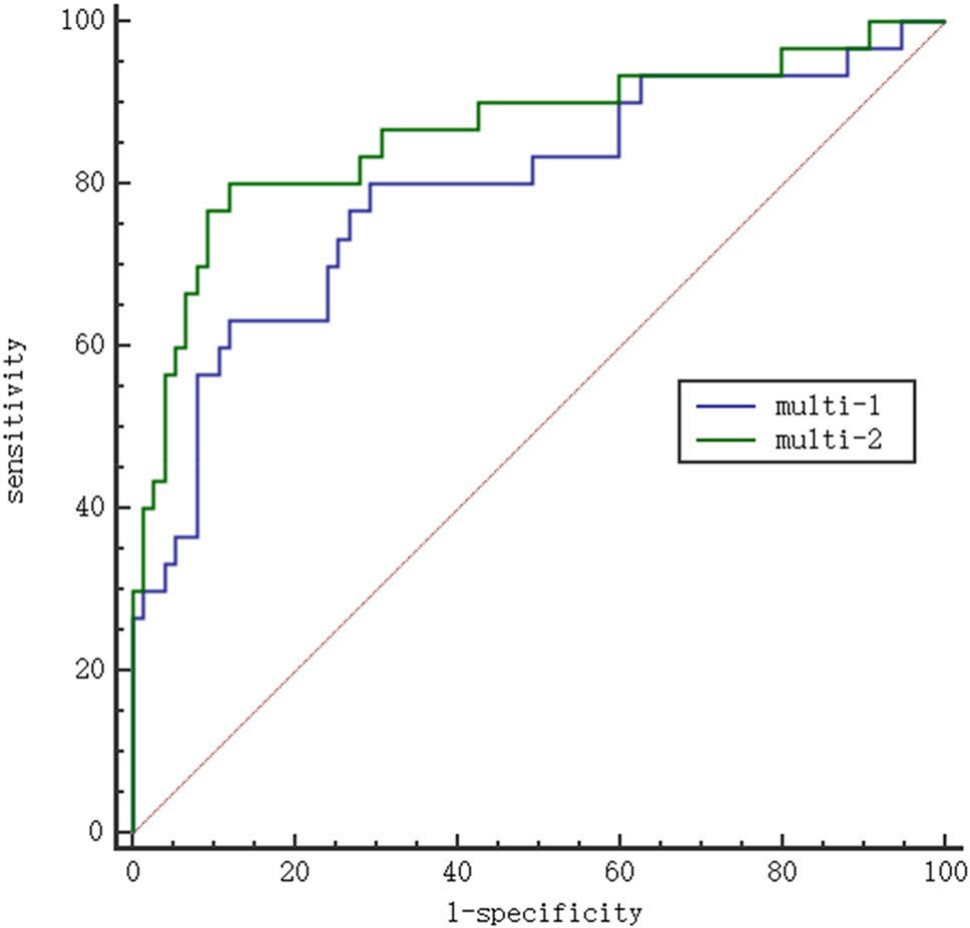
Multi-1 represents the combination of five factors: CT_ART_, IC_ART_, Zeff_ART,_ Zeff_VE_, and NIC_ART_. Multi-2 represents the combination of all factors: CT_ART_, CT_VE_, IC_ART_, IC_VE_, Zeff _ART,_ Zeff_VE_, NIC_ART_, NIC_VE_, λ_ART_ and λ_VE_.

Ten parameters were selected based on the above results and were used for ROC curve analysis for the assessment of their diagnostic efficacy. This showed significant differences between the Luminal and non-Luminal types. The areas under the ROC curves (AUCs) were above 0.64 for all 10 parameters, with five greater than 0.70. These five parameters were then further assessed using ROC curves, finding that the Zeff_ART_ values had the best diagnostic performance for the non-Luminal breast cancer type (Fig. 4) with AUC, sensitivity, and specificity of 0.735, 76.67, and 64.00, respectively (Table 4). Conduct ROC curve analysis on two distinct sets of multi-factor joint diagnostic models (Multi-1 and Multi-2) with AUC of 0.792 and 0.863 (Table4, Fig. 5). Multi-1 represents the combination of five factors: CT_ART_, IC_ART_, Zeff_ART_, Zeff_VE_, and NIC_ART_. Multi-2 represents the combination of all factors: CT_ART_, CT_VE_, IC_ART_, IC_VE_, Zeff_ART_, Zeff_VE_, NIC_ART_, NIC_VE_, λ_ART_ and λ_VE_.

**Table 4:** The diagnostic efficacy of spectral parameters for breast cancer molecular subtypes.

Parameter (unit)	AUC	Sensitivity	Specificity	Threshold
CT_ART_ (HU)	0.714	86.67	48.00	50.2
CT_VE_ (HU)	0.645	46.67	78.67	81
Zeff_ART_	0.735	76.67	64.00	7.56
Zeff_VE_	0.733	60.00	80.00	8.04
IC_ART_ (mg/ml)	0.703	53.33	76.00	0.59
IC_VE_ (mg/ml)	0.666	46.67	82.67	1.36
NIC_ART_	0.700	50.00	81.33	0.06
NIC_VE_	0.694	53.33	80.00	0.36
λ_ART_	0.696	56.67	73.33	0.96
λ_VE_	0.658	73.33	58.67	1.82
Multi-1	0.792	63.33	88.00	0.38
Multi-2	0.863	80.00	88.00	0.32

*AUC* the area under the receiver operating characteristic curves. Multi-1 represents the combination of five factors: CT_ART_, IC_ART_, Zeff _ART,_ Zeff _VE_, and NIC_ART_. Multi-2 represents the combination of all factors: CT_ART_, CT_VE_, IC_ART_, IC_VE_, Zeff _ART,_ Zeff _VE_, NIC_ART_, NIC_VE_, λ_ART_ and λ_VE._

## Discussion

Heterogeneity is a major feature of breast cancer, with significant molecular differences seen in the same pathological cancer type^6^. Treatment of breast cancer is currently mostly determined by the stage and type of the tumor, and is affected by the tumor’s molecular subtype and expression of immunohistochemical markers which determine both the tolerance and efficacy of the treatment^2^. Imaging examinations are commonly used in clinical diagnosis and the preoperative evaluation of breast cancer^13^, including classification and staging and assessment of lymph node metastasis. Mammography and ultrasound imaging are also important and commonly used examination techniques but both result in overlapping images with poor sensitivity and specificity and require highly skilled operators^14^. Furthermore, mammography does not show good repeatability. Magnetic resonance imaging has significant advantages in the assessment of soft tissue such as breasts; combined with DWI technology and gadolinium contrast agent-enhanced scanning, MRI can produce helpful information for diagnosis^15,16^. However, MRI is a time-consuming and costly examination with many contraindications^17^. In addition, MRI has high environmental and patient requirements and the image quality cannot be guaranteed. Dual-layer SDCT can produce images of the breasts, lymph nodes, and lung metastases and can receive a variety of quantitative spectral parameters through the post-processing of a single scan, and thus as high diagnostic value for breast cancer that has been confirmed clinically.

The spectral curve was generated using the different CT values of each tissue under different keV levels. The abscissa contains the 40–200 keV virtual monoenergy level and the ordinate represents the CT value of the tissue at the corresponding energy level. This can reflect the relationship between the absorption coefficient of the tissue and the different energies^18,19^. The spectral curve for breast cancer patients started at 40 keV and gradually flattened as the energy level increased, becoming parallel at about 80 keV. Therefore, we calculated the slope using the 40–80 keV segment and found that λ_ART_ and λ_VE_ differed significantly according to molecular subtype and biomarker expression level. Specifically, ER/PR-negative and HER2-positive patients had higher λ values. This result may be explained by the fact that cancer tissue can take up more iodine than normal tissue. In addition, the new blood vessels formed by aggressive tumors make the attenuation of the spectral curve more obvious. This result confirmed that λ values can indirectly reflect tumor characteristics by reflecting their blood supply.

This study analyzed the diagnostic value of CT values based on conventional images for biomarker expression and molecular subtypes. The results showed that there were significant differences in the mean CT values within each group. Two types of spectral parameters, namely, iodine concentration and effective atomic number, were analyzed further. These parameters (IC_ART_, IC_VE_, NIC_ART_, NIC_VE_, and Zeff_VE_) all showed significant differences between ER-positive and ER-negative, PR-positive and PR-negative, and HER2-positive and HER2-negative tumors. This was consistent with the enhanced CT value which was associated with both greater malignancy and worse prognosis. This discrepancy could be attributed to the vigorous cell growth in high-grade tumors and the greater abundance of new blood vessels, resulting in more obvious vascular perfusion.

This study analyzed the spectral parameters between different tumor subtypes and found differences among the parameters. The non-Luminal type was associated with higher values of the parameters due to its highly invasive nature, particularly, the HER2-overexpression subtype. A possible explanation for this is that the stromal cell density of N-HER2 and TNBC is associated with high vascular permeability^7^. This study further evaluated the diagnostic efficiency of various parameters for the non-Luminal type using ROC curves, finding that Zeff_ART_ had a good diagnostic efficacy with a critical value of 7.56, and multi-factors had the best diagnostic efficacy with a critical value of 0.863. Since the non-luminal type tends to be more aggressive than the Luminal type, the treatment and specific chemotherapy regimens tend to differ considerably. These results show that the molecular subtypes of breast cancer can be identified preoperatively and non-invasively by measuring and analyzing the spectral parameters, which is useful for the formulation of individualized treatment plans for patients.

This study has several limitations. First, the selection and delineation of the ROI was done manually which may result in subjectivity and result in selection bias. Second, this study is characterized as a retrospective study, involving the enrollment of patients diagnosed with breast masses of 4A or higher through ultrasound. It is strongly advised to undergo enhanced CT for a more comprehensive preoperative evaluation. Consequently, the smallest observed mass in this study exceeds 25 mm^2^, thereby resulting in a dearth of research pertaining to tumors of smaller volumes. In subsequent investigations, we intend to broaden our research scope to encompass the analysis of tumors with smaller volumes.

## Conclusion

The dual-layer SDCT enables differentiation of breast cancer biomarker expression and molecular subtypes. Thus, Dual-layer SDCT can be used for preoperative evaluation of breast cancer.

## Data Availability

The raw data required to reproduce these findings cannot be shared at this time as the data also forms part of an ongoing study, but are available from the corresponding author on reasonable request.
